# AJCC 7th edition staging classification is more applicable than AJCC 8th edition staging classification for invasive IPMN

**DOI:** 10.1186/s12957-019-1682-9

**Published:** 2019-08-06

**Authors:** Zhiyao Fan, He Cheng, Kaizhou Jin, Yitao Gong, Qiuyi Huang, Jin Xu, Quanxing Ni, Xianjun Yu, Chen Liu, Guopei Luo

**Affiliations:** 1 Department of Pancreatic Surgery, Fudan University Shanghai Cancer Center, No. 270, Dong’An Road, Xuhui District, Shanghai, 200032 China; 2 Department of Oncology, Shanghai Medical College, Fudan University, No. 270, Dong’An Road, Xuhui District, Shanghai, 200032 China; 3 Shanghai Pancreatic Cancer Institute, Shanghai, 200032 China; 4 Pancreatic Cancer Institute, Fudan University, Shanghai, 200032 China

**Keywords:** Intraductal papillary mucinous neoplasm, Stage, TNM, Prognosis, American Joint Committee on Cancer

## Abstract

**Background:**

Both the 7th and 8th editions of the American Joint Committee on Cancer (AJCC) staging systems have been introduced for pancreatic adenocarcinoma. However, the applicability of these classifications for invasive intraductal papillary mucinous neoplasms (IPMN) has not been systematically examined.

**Methods:**

Patients with invasive IPMN were retrieved from a cohort of 18 geographical sites (1973–2014 varying) in the Surveillance, Epidemiology, and End Results (SEER) cancer registry. The 7th and 8th editions of the AJCC staging were compared. Survival rates and multivariate analyses were computed.

**Results:**

In total, 1216 patients with resected invasive IPMN were included. A major difference between the 7th and 8th systems is the definition of stage IIA (7th, beyond the pancreas without involvement of major arteries; 8th, maximum tumor diameter > 4 cm). The hazard ratio (HR) of stage IIA disease (versus stage IA, HR = 2.33, *P* < 0.001) was higher than that of stage IB disease (HR = 1.48, *P* = 0.087) by the 7th edition classification, whereas the HR of stage IIA disease (HR = 1.26, *P* = 0.232) was even lower than that of stage IB disease (HR = 1.48, *P* = 0.040) by the 8th edition classification. In addition, for the 8th edition staging system, tumor size was not a predictor of survival in patients with resectable tumor > 2 cm (size > 4 cm versus > 2 ≤ 4 cm, HR = 0.91, *P* = 0.420).

**Conclusions:**

The AJCC 7th edition staging classification is more applicable than the 8th edition classification for invasive IPMN.

## Introduction

Intraductal papillary mucinous neoplasm (IPMN) is a rare neoplasm of the pancreas, although its incidence keeps rising in recent years because of the growing use of diagnostic scrutiny [1, 2]. Given the variable risks of malignancy, great importance has been attached to the management of IPMN [3–7]. The risk of malignancy for patients with main-duct IPMN may be as great as 57–92%, whereas the risk for patients with branch-duct IPMN is variable (6–46%) [8]. Mixed IPMN has biological properties similar to main-duct IPMN [9]. Clinical consensuses have been established to manage IPMN, mainly focused on whether surgical resection or close observation should be performed [9, 10]. Obstructive jaundice, main pancreatic duct > 10 mm, and enhanced solid component in the cyst were viewed as the presence of high-risk stigmata of malignancy in the 2017 International Consensus Guideline [9]. However, few studies have focused on the management of invasive IPMN [11–14].

In contrast to non-invasive IPMN, the extent of invasive IPMN has great impact on clinical outcome and management strategies, including whether adjuvant treatments should be administered [11, 12]. Conventional tumor node metastasis (TNM) staging protocols are appropriate to stage invasive IPMN. The American Joint Committee on Cancer (AJCC) 7th edition staging was introduced to stage pancreatic adenocarcinoma in 2010 (Table 1) [15]. In 2016, considering the inapplicability of tumor staging beyond the pancreas in T-stage and the absence of a number of positive lymph nodes in N-stage in the AJCC 7th edition stage classification, the AJCC 8th edition staging classification for pancreatic adenocarcinoma was proposed [16]. Two major modifications were made from the 7th to the 8th edition: (1) primary tumor extension beyond the pancreas was changed to tumor size > 4 cm in T-stage; and (2) N1 (1–3 positive nodes) and N2 (≥ 4 positive nodes) were introduced as positive nodal status in N-stage, and TxN2M0 was included in stage III [15, 16]. Some studies have used the AJCC 7th to evaluate invasive IPMN [11, 12, 14]. However, the biological behaviors of invasive IPMN are different from that of pancreatic adenocarcinoma [12, 14]. Therefore, the clinical applicability of AJCC staging systems for invasive IPMN needs to be systematically validated.

The study was performed to validate the AJCC 7th and 8th staging systems for invasive IPMN by using a large cohort from the Surveillance, Epidemiology, and End Results (SEER) database. The prognostic value of T-stage (primary tumor size and local invasion) and N-stage (nodal status) was also examined.

## Patients and methods

### Patients and data collection

**Fig. 1:**
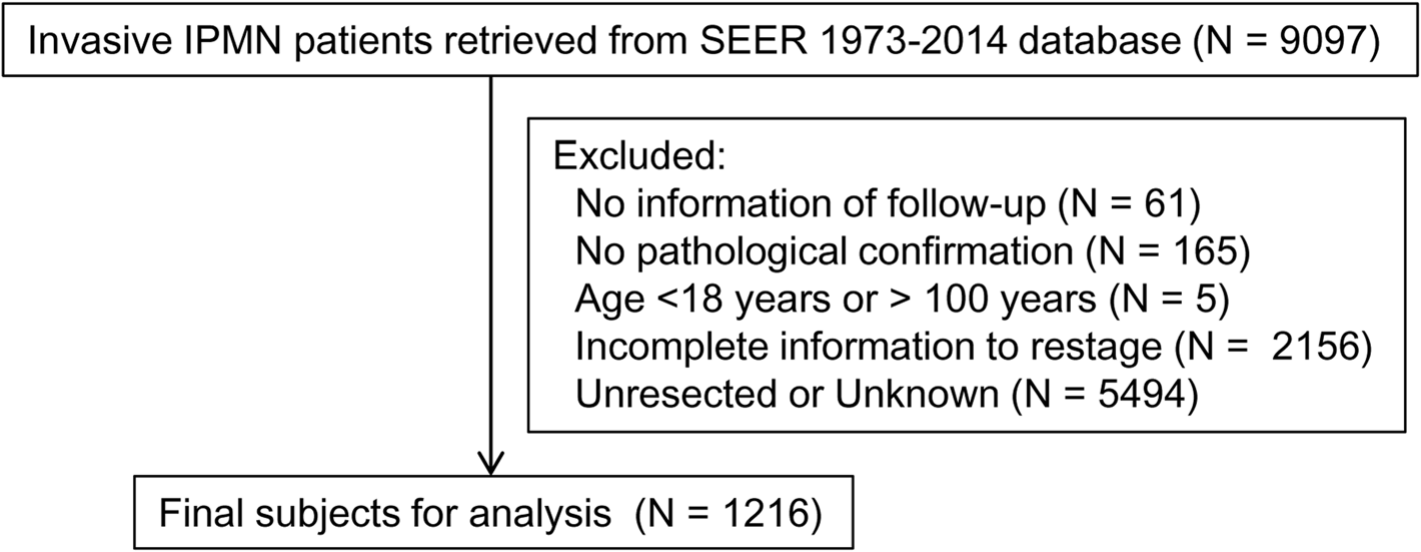
Patient-selection flow diagram of the current study

The SEER database was used to perform the retrospective study. Figure 1 shows the patient-selection flow diagram of the current study. The November 2016 submission was used, including a cohort of 18 geographical sites (1973–2014 varying). The database was retrieved by choosing pancreas as the site recode. The following codes from the International Classification of Disease for Oncology (ICD-O), 3rd edition—8260 (papillary adenocarcinoma), 8050 (papillary carcinoma), 8453 (intraductal papillary-mucinous carcinoma), 8480 (mucinous adenocarcinoma), 8481 (mucin-producing adenocarcinoma), and 8503 (intraductal papillary adenocarcinoma)—were used to identify potential subjects with invasive IPMN [14]. Demographics, including age, gender, race, date of diagnosis, and surgical resection, and tumor variables, including tumor size, location of the primary tumor, and grade, were queried. Tumor size was evaluated by CS tumor size 2004, and node status was evaluated by CS lymph nodes 2004 and “Regional nodes positive (1988+).” All subjects had cytological or pathological confirmation of invasive IPMN. Only cases collected from 2000 to 2016 were included. Patients were excluded if they were younger than 18 years or older than 100 years. Subjects were excluded if they had no pathological or cytological confirmation and/or no follow-up information. Subjects were also excluded if they had insufficient information on the anatomical relationship of tumors to the surrounding vessels (as used in the 7th edition). Subjects who had incomplete information to allow restaging per the AJCC 7th and 8th stages were excluded from the study. For the consideration of accurate staging, patients were excluded if they were unresected or had unknown information of surgical resection. Tumors were graded according to the differentiation of adenocarcinoma (high grade, undifferentiated and poorly differentiated; intermediate grade, moderate differentiated; low grade, well-differentiated). The study was approved by the local institutional review board.

### Statistical analysis

Statistical analysis was performed by STATA 12.0 software (STATA, College Station, TX). Survival time was examined from date of initial diagnostic confirmation until the date of last follow-up or date of death. Kaplan-Meier curves and log-rank analysis were used to analyze the overall survival. Multivariate analysis, controlling by age, sex, race, tumor location, grade, and AJCC stages, was performed using Cox regression modeling. Hazard ratios (HRs) and 95% confidence intervals (CIs) were evaluated. The Aikaike information criterion (AIC) for models containing different staging systems was calculated. A two-sided *p* < 0.05 was viewed as statistically significant. Lymph node ratio (LNR) was calculated by the number of positive lymph nodes divided by the number of examined lymph nodes. The cutoff value of LNR was determined by the receiver operating characteristic (ROC) curve and the area under the ROC curve (AUC).

## Results

### Basic characteristics

In total, 1216 patients with pathologically confirmed invasive IPMN were included (Table 2). The median age of the entire cohort was 67 years (range 18–94), with 41.4% of patients aged ≥ 70 years. The male-to-female ratio was 1.0 (619 men, 597 women). More than 80% of patients were white, 7.8% were black, and the remaining 8.8% were other races. More than half (53.1%) of the patients had tumors located at the head of the pancreas, and 46.9% were at other locations of the pancreas. The median size of primary tumors was 3.5 cm, and 63.1% of patients had tumors larger than 3 cm. Most (74.9%) of the tumors were low or intermediate grade; the rest (25.1%) were high grade. About one third (33.7%) of the patients presented with distant metastatic disease at initial diagnosis.

### Overall survival analysis

The median survival time for the entire cohort was 19.0 months (1-year survival rate, 60.4%; 2-year, 43.4%; 5-year, 26.9%). For patients with localized/regional disease, the median survival time was 34.0 months (1-year survival rate, 79.0%; 2-year, 60.0%; 5-year, 38.1%). For patients with metastatic disease, the median survival time was only 5.0 months (1-year survival rate, 21.9%; 2-year, 9.9%; 5-year, 4.3%). In multivariate analysis, age ≥ 70 years (HR = 1.32, 95% CI 1.14–1.52, *P* < 0.001) and high grade (HR = 1.24, 95% CI 1.03–1.49, *P* = 0.027) were associated with poor outcome assessed by the AJCC 7th stage classification (Table 3). However, only age ≥ 70 years (HR = 1.35, 95% CI 1.16–1.55, *P* < 0.001) was associated with poor outcome according to the AJCC 8th stage classification. In this study, 27.7% of patients had a LNR value higher than the cutoff value of 0.15. LNR was an independent prognostic predictor in both the AJCC 7th (HR = 1.78, 95% CI 1.43–2.23, *P* < 0.001) and 8th edition staging systems (HR = 1.62, 95% CI 1.28–2.04, *P* < 0.001).

### Validation of AJCC 7th and 8th stages

Cross-tabulation of stage distributions are presented in Table 1. Patients classified as stage IB (133 cases) according to the 7th edition were distributed into stages IB (64 cases) and IIA (69 cases) in the 8th edition. Patients classified as stage IIA (190 cases) according to the 7th edition were distributed into stages IA (31 cases), IB (80 cases), and IIA (79 cases) in the 8th edition. Patients classified as stage IIB (314 cases) according to the 7th edition were distributed into stages IIB (207 cases) and III (107 cases) in the 8th edition.

**Table 1:** The 7th and 8th editions of the American Joint Cancer Committee (AJCC) staging definitions for invasive intraductal papillary mucinous neoplasms (IPMN) with cross-tabulation of stage distributions

7th edition				8th edition			
T1	Limited to the pancreas, ≤ 2 cm in greatest dimension			T1	Maximum tumor diameter ≤ 2 cm		
T2	Limited to the pancreas, > 2 cm in greatest dimension			T2	Maximum tumor diameter > 2 ≤ 4 cm		
T3	Beyond the pancreas but without involvement of the celiac axis or the superior mesenteric artery			T3	Maximum tumor diameter > 4 cm		
T4	Involvement of celiac axis or the superior mesenteric artery (unresectable tumor)			T4	Involvement of celiac axis or the superior mesenteric artery (unresectable tumor)		
N0	No regional lymph node metastasis			N0	No regional lymph node metastasis		
N1	Regional lymph node metastasis			N1	Metastasis in 1–3 regional lymph nodes		
				N2	Metastasis in ≥ 4 regional lymph nodes		
M0	No distant metastasis			M0	No distant metastasis		
M1	Distant metastasis			M1	Distant metastasis		
Stage	T	N	M	Stage	T	N	M
IA	T1	N0	M0	IA	T1	N0	M0
IB	T2	N0	M0	IB	T2	N0	M0
IIA	T3	N0	M0	IIA	T3	N0	M0
IIB	T1–3	N1	M0	IIB	T1–3	N1	M0
III	T4	Any N	M0	III	Any T	N2	M0
					T4	Any N	M0
IV	Any T	Any N	M1	IV	Any T	Any N	M1
Edition		8th					
		IA	IB	IIA	IIB	III	IV
7th	IA	124	0	0	0	0	0
	IB	0	64	69	0	0	0
	IIA	31	80	79	0	0	0
	IIB	0	0	0	207	107	0
	III	0	0	0	0	45	0
	IV	0	0	0	0	0	410

**Table 2:** Baseline clinicopathologic characteristics

Parameter	SEER series (*N* = 1216)	
	No.	%
Age, years		
< 70	712	58.6
≥ 70	504	41.4
Sex		
Male	619	50.9
Female	597	49.1
Race		
White	1014	83.4
Black	95	7.8
Others	107	8.8
Location		
Head	646	53.1
Others	570	46.9
Size (cm)^a^		
< 3 cm	313	36.9
≥ 3 cm	535	63.1
Grade^b^		
Low, intermediate	635	74.9
High	213	25.1
AJCC 7th edition		
IA	124	10.2
IB	133	10.9
IIA	190	15.6
IIB	314	25.8
III	45	3.7
IV	410	33.7
AJCC 8th edition		
IA	155	12.7
IB	144	11.8
IIA	148	12.2
IIB	207	17.0
III	152	12.5
IV	410	33.7

*SEER* Surveillance, Epidemiology, and End Results program, *AJCC* American Joint Committee on Cancer

^a^848 patients in the SEER database had data of size

^b^848 patients in the SEER database had grade information

**Table 3:** Multivariate analyses of prognostic factors

Demographic or characteristic	7th edition		8th edition	
	HR (95% CI)	*P*	HR (95% CI)	*P*
Age, years				
< 70	1		1	
≥ 70	1.32 (1.14–1.52)	< 0.001	1.35 (1.16–1.55)	< 0.001
Sex				
Male	1		1	
Female	1.04 (0.90–1.20)	0.572	1.04 (0.90–1.20)	0.574
Race				
White	1		1	
Black	0.91 (0.69–1.19)	0.479	0.89 (0.68–1.17)	0.402
Others	0.80 (0.61–1.04)	0.094	0.79 (0.60–1.03)	0.083
Location				
Head	1		1	
Others	1.10 (0.94–1.27)	0.247	1.08 (0.93–1.26)	0.290
Grade				
Low, intermediate	1		1	
High	1.24 (1.03–1.49)	0.027	1.20 (1.00–1.45)	0.056
Unknown	1.15 (0.97–1.36)	0.111	1.10 (0.93–1.31)	0.253
Stage				
IA	1		1	
IB	1.48 (0.94–2.31)	0.087	1.48 (1.02–2.15)	0.040
IIA	2.33 (1.54–3.51)	< 0.001	1.26 (0.86–1.85)	0.232
IIB	4.31 (2.94–6.31)	< 0.001	2.91 (2.09–4.05)	< 0.001
III	6.08 (3.76–9.83)	< 0.001	4.14 (2.94–5.82)	< 0.001
IV	11.81 (8.13–17.14)	< 0.001	8.95 (6.58–12.18)	< 0.001
C-index	0.75	< 0.001	0.75	< 0.001

*SEER* Surveillance, Epidemiology, and End Results program, *AJCC* American Joint Committee on Cancer

**Fig. 2:**
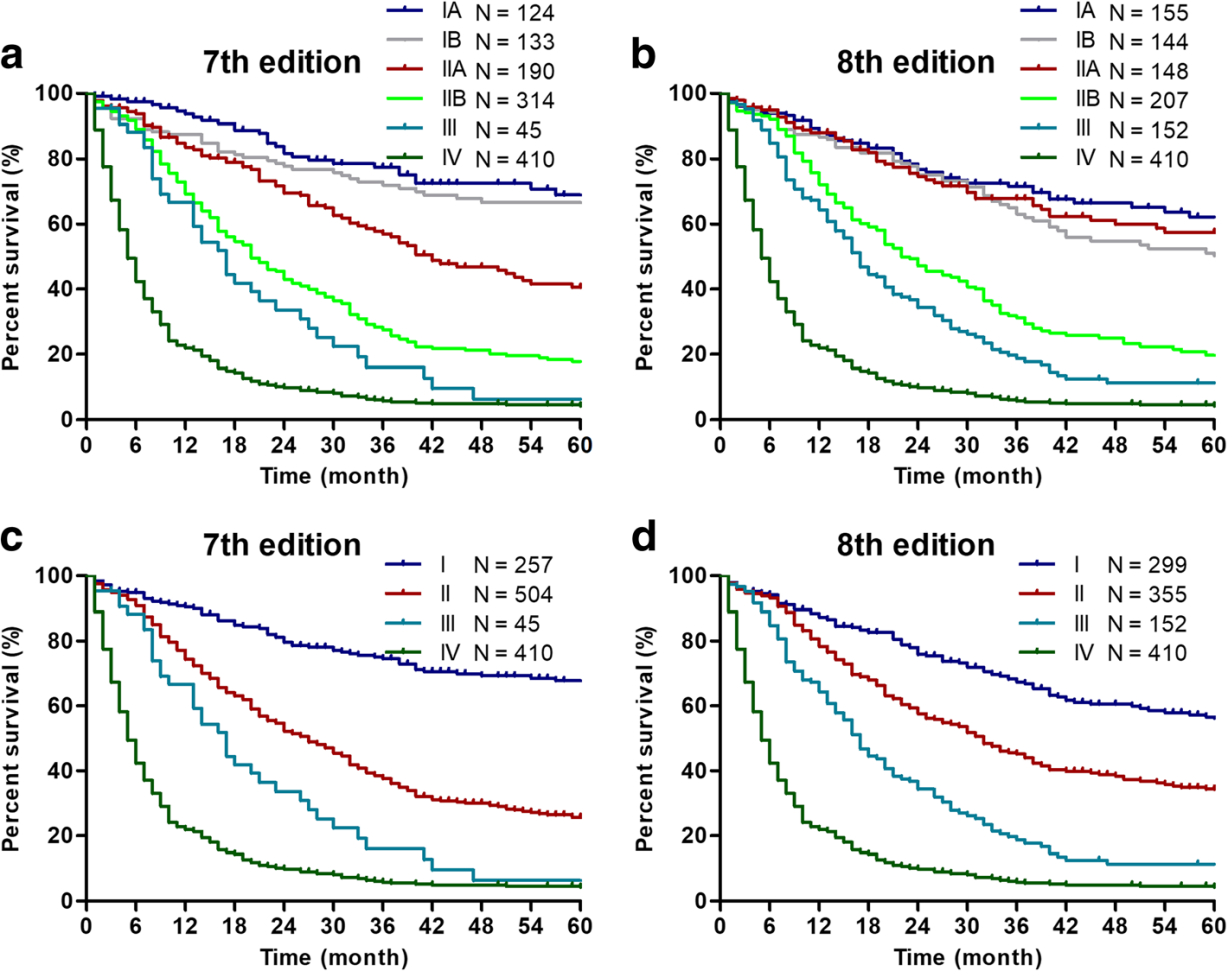
Kaplan-Meier curves of 7th and 8th AJCC staging classifications for patients from the SEER database. Survival curves were well separated by stage, using the 7th AJCC staging classifications (**a**, **c**). However, overlap existed between the stage IB and IIA diseases (**b**)

**Fig. 3:**
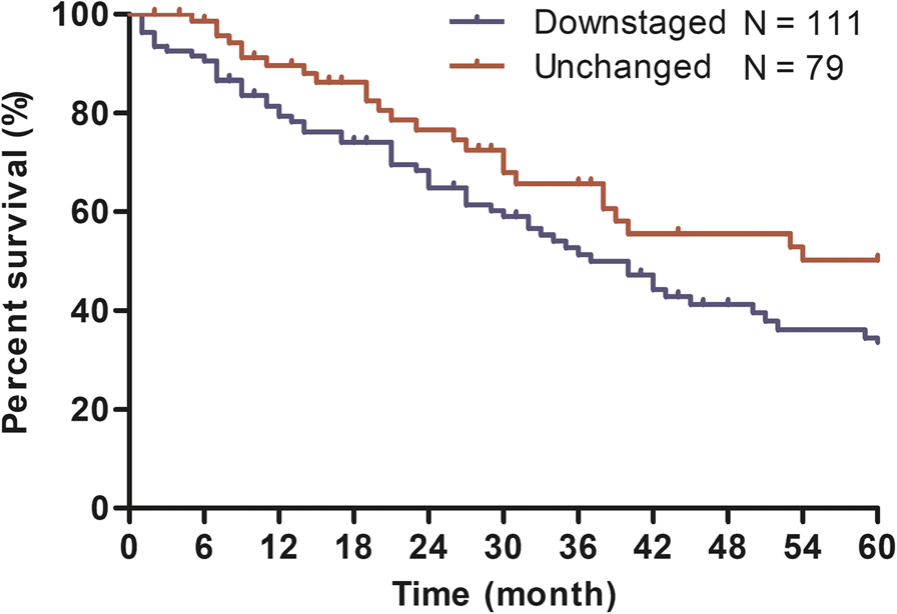
For 190 patients with AJCC 7th stage IIA IPMN, patients with downstaged tumor (111 cases) had better overall prognosis than patients with unchanged disease (79 cases)

For stage classification by the AJCC 7th edition, the HR of stage IIA disease was higher than that of stage IB disease (with stage IA as reference: IB, HR = 1.48, 95% CI 0.94–2.31; IIA, HR = 2.33, 95% CI 1.54–3.51, Table 3) in multivariate analyses. However, for stage classification by the AJCC 8th edition, the HR of stage IIA disease was even lower than that of stage IB disease (with stage IA as reference: IB, HR = 1.48, 95% CI 1.02–2.15; IIA, HR = 1.26, 95% CI 0.86–1.85). Similar results were also obtained by Kaplan-Meier curves (Fig. 2a, b). The AIC values were 1647.98 for the model containing the AJCC 7th edition and 1647.51 for the model containing the AJCC 8th edition. For 190 patients with AJCC 7th stage IIA IPMN, 111cases were downstage into AJCC 8th stage IA (31 cases) and IB (80 cases) and 79 cases remained in stage IIA. Patients with downstaged tumor had better overall prognosis than patients with unchanged disease by the logrank test (*P* = 0.029) and the Kaplan-Meier analysis (Fig. 3).

### Tumor size and outcome of patients with resectable IPMN

Because the major difference between AJCC 7th and AJCC 8th edition stage classifications were N-stage (N0, N1 versus N0, N1, N2) and T-stage (T1–3), the impact of N and T stages on prognoses for patients was further analyzed. Cases with tumor size ≤ 2 cm, T4 (involvement of the celiac axis or the superior mesenteric artery) or M1 (distant metastasis), were excluded from the analysis. For patients with tumor size > 2 cm and resectable tumors, tumor size was not an independent prognostic predictor for all subjects (size > 4 cm versus size > 2 ≤ 4 cm, HR = 0.91, 95% CI 0.73–1.14, *P* = 0.420), nodal-negative subjects (HR = 0.89, 95% CI 0.62–1.29, *P* = 0.553), and nodal-positive subjects (HR = 1.02, 95% CI 0.77–1.35, *P* = 0.913). These findings suggest that the staging classifications in the AJCC 7th edition were more applicable for invasive IPMN than the AJCC 8th edition’s.

## Discussion

In the study, the clinical applicability and prognostic stratification of AJCC 7th and 8th edition staging systems for invasive IPMN were validated using the SEER database. One of the major modifications from 7th to 8th AJCC staging systems is the definition of stage IIA disease (7th, beyond the pancreas but without involvement of major arteries; 8th, maximum tumor diameter > 4 cm). The HR of stage IIA disease (in comparison with stage IA, HR = 2.33, *P* < 0.001) was higher than that of stage IB disease (HR = 1.48, *P* = 0.087) for the AJCC 7th stage classification, whereas the HR of stage IIA disease (HR = 1.26, *P* = 0.232) was even lower than that of stage IB disease (HR = 1.48, *P* = 0.040) for the AJCC 8th stage classification. In addition, for patients with tumor size > 2 cm and resectable tumors, tumor size was not an independent prognostic predictor. These findings suggest that the AJCC 7th edition staging classification was more applicable for invasive IPMN than the AJCC 8th edition staging classification.

Tumor size was a very important predictor of malignancy for IPMN [3, 4]. Size > 3 cm raised the risk of malignant change approximately three times and was one of the worrisome features of imaging in the 2012 International Consensus Guideline [3, 4]. Sub-staging of T1 (1a, ≤ 0.5; 1b, 0.5–1; 1c, > 1 cm) is required to be documented in an international pathologic evaluation and reporting consensus [17]. For patients with resected invasive IPMN, tumor size was found to be an independent prognostic predictor in previous reports and in this study [11, 12, 14]. For example, McMillan et al. showed that tumor size > 2 cm was an adverse prognostic factor for patients with resected invasive IPMN (size > 2 cm versus size ≤ 2 cm, HR = 1.32, *P* = 0.012) [12]. However, for patients with tumor size > 2 cm and resectable tumors, tumor size was not an independent prognostic predictor (size > 4 cm versus size > 2 ≤ 4 cm, HR = 0.91, *P* = 0.420) in the current study.

Previous studies have shown that nodal status was an independent prognostic predictor for patients with invasive IPMN [11, 12, 14]. For example, Wasif et al. demonstrated that positive lymph nodes (HR 1.98, 95% CI 1.50–2.60, *P* < 0.001) was an adverse predictor of survival for patients with resected invasive IPMN [14]. Moreover, both tumor grade and size were predictive of positive lymph status for invasive IPMN [14]. The current study found that either N1 (nodal-positive) in AJCC 7th stage classification or N1 (1–3 nodes) and N2 (≥ 4 nodes positive) in AJCC 8th stage classification were adverse prognostic predictors for patients with resected invasive IPMN, which accorded with previous findings [11, 12, 14].

The current study found that patients with distant metastatic IPMN (stage IV) had a dismal prognosis. For patients with localized/regional disease, the median survival time was 34.0 months (1-year survival rate, 79.0%; 2-year, 60.0%; 5-year, 38.1%). For patients with metastatic disease, the median survival time was only 5.0 months (1-year survival rate, 21.9%; 2-year, 9.9%; 5-year, 4.3%). Therefore, great importance should be attached to early detection of invasive IPMN. In addition, the value of therapeutic methods, including surgical resection and chemotherapy for patients with metastatic IPMN, should be examined.

Similar to pancreatic ductal adenocarcinoma, adjuvant treatments (chemotherapy or chemoradiotherapy) have been shown to have great impact on the prognosis of patients with invasive IPMN [12, 13, 18–20]. Studies have demonstrated that adjuvant radiation was associated with improved survival only in the selected subset of patients with positive nodal status, positive margin, or T3/T4 tumors [12, 13, 18–20]. For example, McMillan et al. [12] collected 1220 patients with invasive IPMN from the National Cancer Data Base (1998–2010) and found that adjuvant therapy was related to improved outcome compared with surgery alone, especially for those with positive margins, positive nodal status, or high-grade tumors. A previous analysis of the SEER database demonstrated that a lower percentage of patients resected for invasive IPMN (35%) had received adjuvant radiation than those with pancreatic ductal adenocarcinoma (42%) [14]. However, the optimal postoperative management of resected invasive IPMN is still controversial for the retrospective nature of previous studies and a majority of studies coming from small institutional series. The effect of adjuvant treatment in the current study could not be assessed for the lack of information about adjuvant treatments in the SEER series.

The AIC values were 1647.98 for the model containing the AJCC 7th edition and 1647.51 for the model containing the AJCC 8th edition. In addition, the C-index for both systems was 0.75. This may be explained by that stage IB in the AJCC 7th edition and stage IIA in the AJCC 8th edition had no statistical significance compared with stage IA in multivariate analyses. These results indicate that both systems should be further improved.

## Conclusions

The AJCC 7th staging classification is more applicable than the AJCC 8th staging classification for invasive IPMN. Tumor size is not a prognostic factor for patients with tumor size > 2 cm and resectable IPMN. Patients with distant metastatic IPMN present a dismal prognosis. However, our study is greatly limited by its retrospective nature, and further prospective studies are needed to confirm our conclusion.

## Data Availability

The datasets used and/or analyzed during the current study are available from the corresponding author on reasonable request.

## Abbreviations

AIC: Aikaike information criterion
AJCC: American Joint Committee on Cancer
IPMN: Intraductal papillary mucinous neoplasms SEER Surveillance, Epidemiology, and End Results
AUC: Area under the ROC curve
CIs: Confidence intervals
HR: Hazard ratio
ICD-O: International Classification of Disease for Oncology
LNR: Lymph node ratio
ROC: Receiver operating characteristic curve
